# Actor-Network Theory and its role in understanding the implementation of information technology developments in healthcare

**DOI:** 10.1186/1472-6947-10-67

**Published:** 2010-11-01

**Authors:** Kathrin M Cresswell, Allison Worth, Aziz Sheikh

**Affiliations:** 1 eHealth Research Group, Centre for Population Health Sciences, The University of Edinburgh, Edinburgh, UK

## Abstract

**Background:**

Actor-Network Theory (ANT) is an increasingly influential, but still deeply contested, approach to understand humans and their interactions with inanimate objects. We argue that health services research, and in particular evaluations of complex IT systems in health service organisations, may benefit from being informed by Actor-Network Theory perspectives.

**Discussion:**

Despite some limitations, an Actor-Network Theory-based approach is conceptually useful in helping to appreciate the complexity of reality (including the complexity of organisations) and the active role of technology in this context. This can prove helpful in understanding how social effects are generated as a result of associations between different actors in a network. Of central importance in this respect is that Actor-Network Theory provides a lens through which to view the role of technology in shaping social processes. Attention to this shaping role can contribute to a more holistic appreciation of the complexity of technology introduction in healthcare settings. It can also prove practically useful in providing a theoretically informed approach to sampling (by drawing on informants that are related to the technology in question) and analysis (by providing a conceptual tool and vocabulary that can form the basis for interpretations). We draw on existing empirical work in this area and our ongoing work investigating the integration of electronic health record systems introduced as part of England's National Programme for Information Technology to illustrate salient points.

**Summary:**

Actor-Network Theory needs to be used pragmatically with an appreciation of its shortcomings. Our experiences suggest it can be helpful in investigating technology implementations in healthcare settings.

## Background

### Studying IT implementations in healthcare settings

Information technology (IT) is increasingly being used to facilitate the communication of information across healthcare teams and groups with the aim to make the delivery of care safer and more efficient [1,2]. Several countries worldwide are therefore now committed to the increasing use of IT in healthcare settings [3]. These interventions need to be evaluated and in this respect the importance of theoretically informed investigations of these new technologies, in order to maximise the effectiveness of implementation, has repeatedly been highlighted [4-6].

The underlying assumption underpinning the introduction of IT in healthcare is that improvements of information flow will translate into improved quality of care [7]. However, transmission of information through IT is not straightforward as inputs are often transformed into unpredictable outputs [7]. This is especially true for the multifaceted healthcare environment where many different groups use various technologies in complex ways.

Studying IT in healthcare is therefore in many ways like trying to "hit a moving target"; it requires different social theories than were present before its rise. On the one hand, there is the need to simplify reality, but the flip side of this is that this simplification should not be to the extent that this masks the fine nuances that characterise this complexity [8]. Law and Mol argue that researchers need to acknowledge this challenge, whilst paying attention to multiple scenarios that reveal different stories, which may relate to each other. The aim is to try to find out how these different stories/worlds are related, which is in turn assumed to lead to insights into aspects of the complex picture one is studying.

Several specific approaches have been developed to study humans and their interaction with technology in organisations. One of the most commonly cited theories - because it pays close attention to this complexity - is the Socio-Technical Systems (STS) perspective. A socio-technical system can be described as a system (e.g. an organisation such as the National Health Service (NHS)) where technical dimensions (e.g. a specific IT system) and social dimensions (e.g. attitudes and relationships of stakeholders) are interrelated. The extent to which these shape, fit and complement each other is believed to be important in determining how the system functions [9,10].

Actor-Network Theory (ANT) draws on the STS perspective. In this paper, we reflect on the potential contributions of ANT and its practical applicability to evaluations of IT in the healthcare setting. We illustrate the key issues through reflecting on our ongoing investigation of the introduction of electronic health record (EHR) software in specialist care settings as part of England's National Programme for Information Technology (NPfIT) (Table 1). In so doing, both the benefits of using this approach and the challenges we experienced will be discussed. This manuscript will touch upon some emerging findings in our ongoing research - the full integration with our results will be the subject of a separate piece once our data collection has been completed.

**Table 1:** Summary of an ongoing investigation of the introduction of electronic health record (EHR) software in specialist care settings as part of the England's National Programme for Information Technology (NPfIT)

**Background**: As part of the English National Programme for Information Technology, trusts are encouraged to implement centrally procured Electronic Health Record Software. However, the introduction of such systems in healthcare settings radically transforms the nature of healthcare professional work practices.
**Aim**: The overall aim of the research is to explore early views and experiences of healthcare professionals using the new EHR system in selected secondary and community care settings.

**Methods**: We are conducting a mixture of semi-structured interviews with various stakeholders including implementation team members, healthcare professionals and other users of the software in selected early implementer trusts. This is complemented by observations of users.

## Discussion

### A very brief introduction to Actor-Network Theory

ANT's main feature is its focus on inanimate entities and their effect on social processes. An actor is thus defined as the "source of an action regardless of its status as a human or non-human"; this is a radical notion in that it contests that inanimate things (e.g. such as technology) can also have agency [11-14]. An actor can however only act in combination with other actors and in constellations that give the actor the possibility to act - this is because reality is assumed to be actively performed by various actors in a particular time and place [8,13,15,16]. Thus inherent to ANT is a move away from the idea that technology impacts on humans as an external force, to the view that technology emerged from social interests (e.g. economic, professional) and that it thus has the potential to shape social interactions [14].

ANT has its own epistemological and ontological position, in essence considering the world as consisting of networks [17]. These networks can include humans, things, ideas, concepts - all of which are referred to as "actors" in the network. Tracing of associations or relationships between network components (or actors) is a key activity in ANT [18]. ANT assumes that the sum of non-social phenomena can account for something that is social as a result of constellations of human and non-human actors constituting the network. It follows then that the ANT approach is agnostic with respect to the debate which has divided many sociologists in that it neither asserts that everything is socially constructed (social constructionism) nor that everything is pre-existent (realism) [16].

The central idea of ANT is to investigate and theorise about how networks come into being, to trace what associations exist, how they move, how actors are enrolled into a network, how parts of a network form a whole network and how networks achieve temporary stability (or conversely why some new connections may form networks that are unstable) [13,19,20]. The aim is to gain detailed insights into how social effects such as power come into being [20,21]. This is vividly illustrated by Law through a parable in which he describes how objects such as a big office, a computer and a phone can serve to create the manager in an organisation as the source of power [22]. The manager studied in isolation (as a person or "naked ape" as Law calls him i.e. without objects), as opposed to as part of a network, is viewed as relatively powerless.

ANT assumes that if any actor, irrespective of its position, is removed from or added to the network, as is the case if technology is introduced into an organisation, then the functioning of the whole network will be affected [13,23]. However, networks are constantly evolving as social reality is assumed to be both complex and fluid (further discussed below) [24].

The composition of networks tends to become particularly apparent when things in a system go wrong; conversely, these inter-connections tend to be hidden when things are working smoothly [14,17,21]. A key task for the ANT researcher is to explore how local networks are ordered and re-configured over time [14,17].

Intermediaries and mediators can form relationships between actors [18,25,26]. The difference between the two is that the outputs of intermediaries can be easily predicted on the basis of their inputs (a black box). In mathematical terms, the assumption here is that X directly causes Y. Mediators, on the other hand, transform inputs into unpredictable outputs. This means that they can also transform actions, making something happen that is not necessarily related to what set it into motion [21]. In mathematical terms, the effect of X on Y is in this case influenced by some other variable such as Z. ANT assumes that the social world consists of many mediators, which tend to be the focus of analysis as they impact on social outcomes in often unpredictable ways, and very few intermediaries [20].

Since its conception in the 1980s, Latour, Callon and Law have remained the most influential thinkers in this field. They have as a result often been the butt of fierce criticisms, particularly relating to ANT's radical ontological assumptions. Challenging criticisms and intellectual exchanges have led to some evolution of the ways in which ANT is formulated [24], but it essentially remains a view of the world as made up of networks in which objects can have an important role in shaping social relations [20,21]

### How can ANT inform the study of IT implementations in healthcare settings?

Purist applications of ANT remain uncommon and even when used the subject of considerable debate [13,25,26]. A case for such "authentic" ANT studies (i.e. those that adhere to the strict and original principles of ANT without modification) continues to be made [27], but we believe that such approaches are unlikely to be the most helpful way to study the introduction of technology in complex healthcare settings due to a number of factors discussed below. Rather, we focus on examining the value of the pragmatic application of the ANT approach in studying IT implementations in healthcare settings.

In broad terms, there are at least two ways in which the ANT-informed approach to studying IT implementations in healthcare settings can be helpful - conceptually and practically. To illustrate this, we will draw on existing examples of the use of ANT in health services research to study technology introduction [11,14,25,28-34], and our own ongoing work. In doing so, we will also describe challenges we have faced in using the approach and outline ways in which we have tackled these.

### Conceptual value of the ANT-informed approach

#### Fluidity of reality

Conceptually, the ANT approach can be valuable in helping researchers to appreciate the complexity and fluidity of reality, which may be neglected by research approaches assuming a more linear and causal approach to studying IT implementations [15,24,35]. As a result, ANT helps to conceptualise how different realities are experienced and enacted by different actors, resulting in a more nuanced picture of the dynamic relationships between different actors without neglecting their inter-relatedness. This is important when considering the fast-moving and ever-changing area of healthcare itself, and particularly so in relation to government-led change initiatives and resulting changes in power relationships [36].

In this context, several authors have illustrated how ANT can be a useful tool for exploring changing power relationships in relation to both healthcare reforms and IT introduction [20]. For example, Lowe drew on ANT to explore changes resulting from a health reform in New Zealand [37]. Here, the organisation, in itself a network, was assumed to be situated in a larger network of politics and other organisations. Managers were enrolled and empowered by the government to achieve the aims of the reform. These managers, in turn, had to enrol individual groups within the organisation so that established networks could be re-organised. The authors describe how new government policies focusing on quantification, emerging from governmental concerns about inefficiencies in the health service, resulted in changes in the position of different groups in healthcare organisations over time (e.g. from doctors assuming dominant positions to an increasing influence of nurses).

We have observed similar shifting power relationships in our ongoing research of EHR introduction as part of the NPfIT. In this case, software has been centrally procured by the government from a small number of commercial suppliers. As a result, both hospitals and end-users needed to be enrolled to implement and use the software - thus a similar situation to that referred to in Lowe's study described above [38]. However, changes in governmental structures combined with budget cuts meant that the national strategy has changed over time to include increased flexibility and local choice in the way EHR software is implemented [36]. Consequently, power relationships have shifted over time from so called "top-down" implementation strategies led by the government, to increased input and choice of local hospitals and users [36]. As a media article, published by eHealth Insider in 2008 reads [39]:

"Mr O'Brien pointed out that the party has commissioned an independent review of NHS IT, led by Dr Glyn Hayes. He also indicated that, in future, there would be far more focus on local systems, built and linked by standards. "Interoperability is the key to achieving those links. That is our first principle," he said."

The examples referred to above relate to studying the fluidity of networks and shifting power relationships from a macro perspective. However, they can also be studied from a micro perspective. A way to address this may be by studying networks longitudinally and then comparing how different constellations of actors change over time. It is important that these changes in the network are investigated and documented as they can help to inform future implementations by giving an indication of where to focus efforts and which temporary effects can be expected to attenuate over time. For example, during early adoption of a particular technology, certain problems may be short-lived and attenuate with increased use. Indeed, we have found this to be the case in the context of our study, as over time users get more proficient in using the software and to some extent find ways of accommodating it within their existing work practices. This may, for example, take the shape of preparing for a clinic whilst waiting for the computer to start up.

#### The active role of objects

The ANT approach can also help to guard against simplistic assumptions in relation to the role of objects in shaping social realities. They are no longer viewed as passive "black-box" containers of information, but as playing an active role that is determined by their position in the ever-changing network. Therefore, the essential value of ANT lies in challenging assumptions of separation between material and human worlds [21,40,41]. This conceptualisation provides a good tool for investigating complex relationships between human and non-human actors in which boundaries in relationships are blurred [20].

One of the most prominent writers illustrating this active role of objects in healthcare settings is Berg, who has analysed the active role of the medical record in mediating social relationships between healthcare staff [42]. He describes how the record structures medical work through the processes of reading and writing, how it coordinates care across professional boundaries and also how it contributes to sustaining power relationships between human actors. These analyses provide a helpful insight into the complexity of different forces at play, illustrating how artefacts can transform care by influencing relationships between human actors. The extract below vividly illustrates this (Table 2).

**Table 2:** An extract from Berg describing the active role of the medical record in mediating relationships

*Through practices of reading and writing, the medical record functions as a constitutive element of current medical work. It enters into the 'thinking' processes of medical personnel and into their relations with patients and with each other. It helps to shape the form the patient's trajectory takes, and it is actively involved in the transformation of the patient's body into an 'extension' of the hospital's routines. The record, as a distributor and collector of work tasks, allows a high level of complexity in the organisation of work - yet its own functioning is constantly amended, repaired, and played upon by the same staff members whose work practices it transforms.*
Extract from Berg 1996

The active role of the record in mediating social relationships has also been helpful in conceptualising our own research. Here, conventional paper systems are replaced by new EHR software and this has radically changed the way the healthcare team operates. For example, in one of our study sites, before the system was introduced, nurses were informally ordering x-ray requests on paper forms, often pre-signed by clinicians. This was no longer possible with the new system due to restricted access rights for nurses, who did not have the necessary training. As a result, consultants were forced to order x-rays themselves.

#### Acknowledging multiplicities

ANT's focus on fluidity also means that it acknowledges that reality is not predictable and that multiple realities can coexist, with reality being actively performed in different contexts and by different actors [15]. Social effects are assumed not necessarily to have any specific origin, but rather to emerge from these multiplicities. It follows that things (or actors, or tools) are what they are depending on the context in which they are embedded and used. This means that they can also be multiple, but these multiples are in some way related and can overlap [15].

Inherent to the notion of multiplicities is that these can be conceptualised in multiple ways and that they are, as a result, difficult to study. We consider below some ways to approach this in relation to different attributes, roles and perspectives of actors.

Firstly, the notion of multiplicities helps to deal with different attributes of both human and non-human actors. This is an oft-cited criticism of the ANT approach: i.e. that it fails to take into account human intentions, interests between different groups, morals, learning, backgrounds, routines, culture and previous experiences of human actors [16,20,21,40,43]; and inherent attributes of objects in line with their history that shapes their role in the network [16,21]. Therefore, many have highlighted the need to recognise that different actors can play multiple roles in multiple networks at multiple time points [3,20,22,43].

Secondly, the notion of multiplicities can also help to conceptualise different roles of actors. Singleton and Michael give a helpful example of how the notion of different roles of human actors could be conceptualised in the healthcare context referring to a case study of a cervical screening programme [43]. The authors describe that when this was introduced, general practitioners (GPs) seemed to have two roles, including that of an enabler (enrolling others into the network) and that of a critic (threatening the stabilisation of the network). In addition, acknowledging the multiplicity of networks themselves, the screening programme was also described as only part of a larger network and only a small part of the GP's role in general.

Thirdly, the notion of multiplicities can help to conceptualise different perspectives of human actors and forms of non-human actors. For example, Bloomfield outlines tensions between those who manage the introduction of the IT system (e.g. managers and policy makers) and those who need to use it in their everyday work [7,44].

In our ongoing study, we have similarly found that the EHR can in itself be multiple as it tends to mean different things to different stakeholders and in different contexts. For some, it takes the form of a vehicle of managerial control that keeps them from carrying out their daily care-giving tasks, whilst for others it is exactly this type of control that makes the system so valuable, for example by allowing monitoring activity levels and organisational outputs.

#### Exploring micro processes in a complex environment

ANT does not *a priori *divide the world into micro and macro contexts or attribute agency to either individuals or social structures [16,20,37]. Rather, agency is assumed not to be limited to individuals, objects or social determinants, but as emerging as an effect of the interactions of network components [45]. These components theoretically consist of the same basic building blocks [46]. ANT therefore focuses on examining the micro context (e.g. individuals directly interacting with technology) and uses findings to draw conclusions about the macro context (e.g. the political environment in which individual practices are situated) [20,47]. This is achieved by incorporating actors from both contexts into the same network [20,21].

Complexity is, however, difficult to study and it is important to recognise that one will never be able to capture the full picture of social reality [8]. Nevertheless, ANT can help researchers to "zoom in" on the way networks consisting of human and non-human actors are formed at any point in time. This focus on micro contexts can help to shed light on the subtleties of social reality and thereby help to make inferences in relation to wider social processes ("by zooming out").

**Figure 1:**
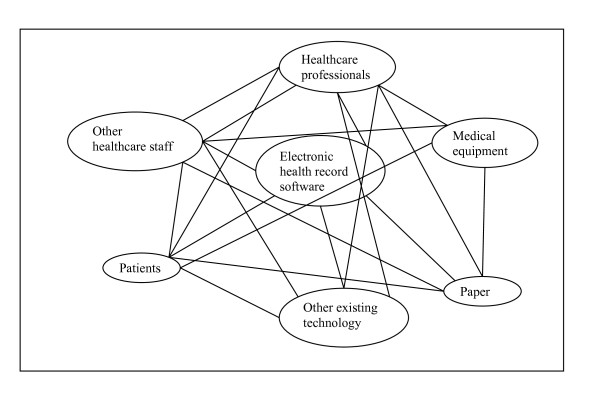
A simplified illustration of a potential network in relation to the introduction of an electronic health record system

From a micro perspective, healthcare technology may be viewed as a new component added to an established network consisting of healthcare staff and existing objects (e.g. paper, medical instruments, other information systems). Figure 1 illustrates in simplified schematic terms how such a network may, for example, look in relation to the introduction of an EHR system. The integration of the new EHR system requires the formation of new connections and other more established network components to re-organise around this new actor and ANT can help to gain a deeper insight into the processes involved. This can then result in recommendations of how to make the new network - i.e. one now including both humans and technology - more stable and in so doing facilitate the effective integration of the technology into the healthcare environment.

In doing so, barriers such as difficulties integrating the new software into work practices of users can be identified. This may then help to explain why an implementation was slower than expected or came to be labelled as a "failure" by some. Studies by Berg [42] (mentioned above), are good examples of investigations these micro-processes. Similarly, we have found issues such as difficulties with integration into work practices in our ongoing study. This, in turn, was perceived to impact on implementation timelines, as users found it difficult to get used to new software that radically changed the way they were used to working.

### Practical value of an ANT-informed approach

The ANT approach can also have practical relevance for investigating the introduction of IT in healthcare settings. It can be used as a tool for sampling by focussing on relevant informants that are related to the technology in question. In this context, ANT has been used in combination with multi-sited ethnography [48,49]. This is an innovative approach that focuses on studying multiple locations, as opposed to the in-depth study of a single local setting that characterises traditional ethnography [48,50]. Multi-sited ethnography aims to gain an insight into both local contexts and the wider social system in which these are situated [48,50]. These local contexts (or sites) are, although different, to some extent assumed to be related and purposefully chosen, which fits in well with Latour's concept of associations [48,50,51]. It is thus important to examine connections, impacts and local differences over time in the face of some common phenomenon [48,49].

Drawing on a combination of ANT and multi-sited ethnography, Bruni [52], describes an ethnography of the electronic clinical record in an Italian hospital. Although this study did not go beyond the boundaries of the hospital, it was argued that the electronic record helped to facilitate the definition of the ethnographic space by following the software, and tracing its relationships with other actors over time. The paper described that the software was still a new addition to the network "negotiating" its right for existence in the organisation. Effective integration of IT will only happen, it is argued, if it can be absorbed into the network of other existing actors. In a similar vein, Mol describes how to do an ethnography of a disease[53]. It is suggested that this approach can help to capture the complex ways the different parts of the body and the disease are enacted by different actors and in different places and at different times.

**Figure 2:**
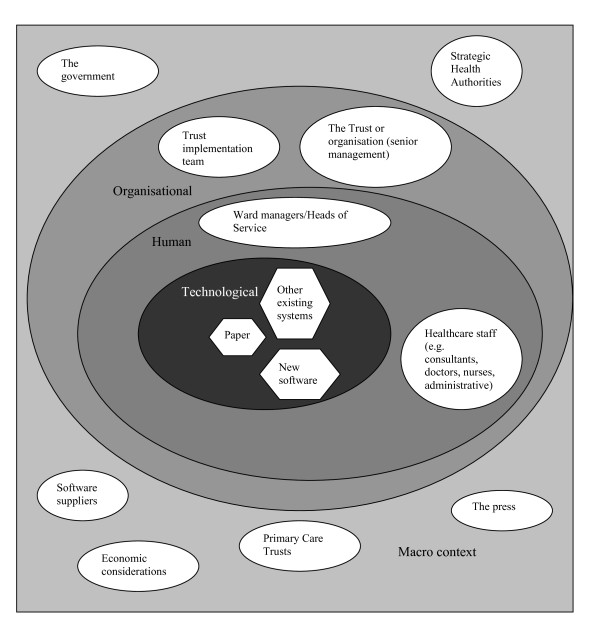
A simplified illustration of a network incorporating micro and macro contexts using the introduction of an electronic health record system as an example

Using ANT in combination with multi-sited ethnography can help to focus data collection and inform strategic decisions throughout the conduct of the research. Figure 2 illustrates how this may be conceptualised in relation to investigating the introduction of electronic health record software. By following the technology as an actor and tracing its connections with various other actors organically, this approach can help to understand different perspectives that constitute the components of the network and hold it in place. In this context, ANT can serve as a roadmap, a way of expressing in simple terms (network terms) the complexity of what is "out there", and as a way of making sense of things by investigating how they are connected.

### Potential challenges of using an ANT-based approach

As with all other approaches to social theory, in attempting to answer the question of how social orders are created and maintained ANT faces epistemological, ontological and methodological challenges. Some of these will be outlined in turn, in line with ways in which these have been addressed in our ongoing research.

#### Is ANT a method or a theory...and does it matter?

In fact, it may be ANT's practical applicability that has some led to conclude that "ANT's main shortcoming is its being everything but a theory", a criticism which has been partly attributed to its (allegedly inappropriate) naming [13,21]. The essence of this criticism is that the approach is too descriptive and failing to come up with any detailed suggestions of how actors should be seen, and their actions analysed and interpreted [21,26,54,55]. It has therefore been proposed that ANT may be best used in a combination with other theoretical approaches, especially in relation to analysis and interpretation [47,56,57]. However, a detailed discussion of combining ANT with other theoretical lenses is beyond the scope of this paper and will be explored in due course in a follow-on paper.

Nevertheless, it may be useful to consider the traditional notion of theory to explore this issue further. A theory should be able to explain "how and why" things occur by exploring their relationships [54]. Describing how things occur is straightforward using ANT, but why things occur poses a challenge. Other problems facing ANT are that it is difficult to test with empirical evidence as it is very broad and hence difficult to refute. It can therefore serve to aid explanation, and provide a vocabulary for interpretation [20,21]. It has, however, limited capability in developing empirically verifiable evidence.

Hence, we have found it helpful to view ANT as a something between a theory and a method, or more precisely as an analytical technique where the researcher follows actors and tries to understand what they do whilst constantly questioning often taken-for-granted characteristics of actors and accepting the flux and changing nature of reality [21,40]. In so doing, ANT has been said to be "telling tales about how the world cannot stop transforming" [40]. Here, the approach can be useful in helping to frame the research question, guide data collection and theorise about potential explanations [58].

Qualitative research, the main method of data collection in ANT, is generally more suited to theory development than to hypothesis testing, shifting the analytical focus to sense making activities, negotiation, differing actor perspectives and emerging effects. Yet from our own experience drawing on the approach, it is particularly important for researchers not to loose sight of the wider study aims as ANT studies can be prone to getting lost in detail. For example, detailed descriptions of individual work practices and ongoing examination of how different groupings conceptualise the EHR in different ways, without attempting to relate this to other relevant factors and the study questions, may be unhelpful, resulting in a lack of practical suggestions for improvement.

#### Researching a fluid reality and multiplicities

The challenge for researchers dealing with multiplicities and a fluid reality is to achieve a balance between the focus of the investigation and acknowledging that multiple different realities can exist without letting these differences mask the complexity of relationships. The result is that ultimately a choice needs to be made between which context to study and which part to focus on (without neglecting the whole picture) as one can not possibly capture everything. This is likely to be determined by the research question, practical constraints and the focus of the study. Conceptually, it may therefore be useful to view networks as consisting of several sub-networks. For example, part of the larger network of a national EHR implementation may be healthcare professionals using a particular piece of technology in a particular ward. One could then examine how these different networks align or fail to align (e.g. across different wards) and how they are positioned in relation to each other and larger networks (e.g. the hospital, the historical, cultural, political environment) [43].

This leads to another problem. Even if the focus of the investigation is on the micro context such as, for example, individual healthcare practices and their relationship with IT, the number of potential actors is conceptually infinite, leading to the question of what to include (or exclude) in the network as for practical reasons analysis and data collection cannot continue forever [16,21]. It follows that at some point researchers need to make decisions in relation to where to start and stop data collection. Whilst taking into account all relevant actors (i.e. those that play a role outside the immediate environment such as, for example, the hospital in influencing the functioning of the network), the primary focus should be on answering the research question [20]. This may involve focusing on a particular network or aspect of a network in more detail (e.g. the micro context if investigating work practices) and is particularly relevant in health services research as time and resource constraints often limit the breadth and depth to which networks can be examined.

#### The positioning of humans and non humans

Not surprisingly, the equal positioning of human and non-human actors in ANT has stimulated debate. It is not the purpose of this paper to delve into detail as to how the two differ, but in the course of our research, we have encountered one issue that ANT has particular difficulty accounting for: the researcher, which warrants further exploration here.

ANT views the researcher as agnostic (or detached), typically eliciting textual or verbal data from human actors through qualitative interviews and observations. Here, humans are both informants (i.e. actors that generate accounts) and interpreters (i.e. the researcher as interpreting associations and components of the network).

We have however conceptualised the researcher as part of the network, as it is hard to imagine the existence of a truly detached observer as (s)he always comes from a particular position in time and space and thus must play an active role in eliciting and constructing ANT accounts [20,21,43,59]. Researchers also have considerable influence on how actors and informants are selected and this too needs to be taken into account [20].

This issue is part of a larger epistemological debate in social science rather than being particular to ANT, but is nevertheless worth considering as researchers utilising the approach will be likely to face these questions. In line with this, health services researchers need to acknowledge that they will be part of the network and will transform it as much as it will transform them as relationships are formed throughout the research process, particularly if this involves qualitative methods. Researchers should be explicit about this involvement and show an awareness of how accounts are produced and how choices are made [20].

## Summary

The increasing scale of computerisation of modern healthcare highlights the need for a more sophisticated view of relationships between humans and objects as technologies become ever more complex. In this context, ANT has stimulated academic debate with its radical approach to conceptualising agency and relationships between humans and objects. The present paper has outlined how the approach can usefully inform evaluation research on the implementation of IT in the healthcare setting and has given some recommendations on how to conceptualise this. The main value of the approach lies in a more sophisticated appreciation of the fluid and multiple nature of reality, the view of the active role of objects in shaping social relationships, and a theoretically informed approach to guiding sampling and data collection. Challenges of the approach and potential ways to address these have been outlined and illustrated using EHR implementation as an example throughout the paper. A summary of both the contributions offered by and challenges inherent to the ANT approach in relation to our ongoing research is given in Tables 3 and 4 below. On a more general note, we conclude that, in relation to any methodological approach certain assumptions and choices have to be made by individual researchers and often depend on ideological circumstances. These choices can also mean combining different approaches, without the need to strictly follow one or the other. It is important to keep in mind that methodology cannot resolve the higher epistemological debate, but it can help to understand social processes and how different issues materialise in different settings.

**Table 3:** Potentially valuable contributions of the ANT approach in a study exploring the introduction of electronic health record software

Key notion	Valuable contributions of this notion	Implications for our study	How the study would look if it was not informed by ANT
Translations	Detailed insight into the complexity of different forces at play when artefacts are introduced in a new context - this can also help to inform sampling considerations	Insight into how the software (which was designed by computer scientists) is integrated into the healthcare environment	Might be tempted to neglect the design context and examine the adoption context in isolation
Active role of objects	How objects can actively transform established practices by influencing the way human actors are associated	The software is viewed as actively transforming the way care is delivered rather than being a relatively passive piece of equipment	Software may be viewed as passive, which may lead to underestimating its influence
Analytical method and theory development	As a conceptual tool to guide the research process, frame the research questions, collect and interpret data and theorise about potential explanations	Focus on a certain technology as a case and sampling different human parties associated with it, notion of networks can help to conceptualise connections and active role of objects can help to theorise about potential outcomes	Sampling may neglect potentially important actors, may result in a limited and a-theoretical approach
Generalised symmetry	Can help investigators to resist imposing a priori differences between actors	Helps to recognise that objects can create unpredictable outputs and have agency	Prior assumptions of dualism between humans and objects may distort the analysis
Enrolment	Can help to explore how different parties/actors are enrolled into a network and how relationships are formed over time	Helps to map out interests of different parties and how the most powerful (e.g. managers) try to enrol the users in adopting the software	May not be able to capture the different effects and stages of change in detail

Flux and changing nature of reality	A tool for exploring how complex relationships between actors and effects come about through movements in the network (e.g. power relationships, social effects)	Helps to conceptualise how change is a process and context dependent	A rigid view of reality may be too simplistic and mask the complexity of change

**Table 4:** Additional factors to consider when using a modified ANT approach

Methodological issue	How this may be addressed	Implications for our study
ANT does not a priori divide the world into micro and macro contexts or attribute agency to either individuals or social structures	Broader contextual factors should be taken into account and may be viewed as other parts of the network	Political, cultural and economic environments are important to consider when examining the introduction of the software
The number of actors in the network is potentially infinite	Researchers need to make rigorous and pragmatic decisions of where (and from whom) to start and where to stop data collection. The primary focus should be on answering the research question.	Although the focus may be on exploring changes to work processes, views from other relevant stakeholders such as implementation team members, developers and governmental stakeholders may also be sought
Different actors can play multiple roles in multiple networks at multiple time points	May be useful to view networks as consisting of several sub-networks and as changing over time	Can examine how different networks align or fail to align (e.g. use of systems across different wards), how they are positioned in relation to larger networks (e.g. the hospital, the historical, cultural, political environment) and how networks change over time (e.g. comparing early and later implementation stages)
ANT is too descriptive and fails to come up with any definitive explanations or approaches of how exactly actors should be viewed and analysed	Important not to lose sight of the wider study aims as purist ANT can be prone to getting lost in detail	The focus of the study is on examining the changes in work processes as a result of the introduction of the software and all other activity should centre around this primary research question
A truly detached observer does not exist as he/she always comes from a particular position in time and space and plays an active role in eliciting and constructing accounts	Researchers need to be pragmatic and acknowledge their involvement through reflexive accounts	It is important to keep a field journal and reflexive notes throughout data collection and analysis

Human accounts and often those of the most powerful are privileged offering little insight into the material world	Need to recognise individual differences between humans and acknowledge that artefacts have attributes and a history	Take into account differences arising from different actors by being explicit about their positioning and the attributes of the technology arising from different historical constellations

## Abbreviations

ANT: Actor-Network Theory; EHR: electronic health record; IT: Information technology; NHS: National Health Service; NPfIT: National Programme for Information Technology; STS: Socio-Technical Systems

## Competing interests

The authors declare that they have no competing interests.

## Authors' contributions

KC is guarantor. This paper is part of KC's PhD investigating the impact of Electronic Health Records on healthcare professional work practices. AS and AW conceived the idea for this paper and contributed to interpreting the evidence and drafting the manuscript. KC identified and reviewed the literature and led the drafting of this paper. All authors have read and approved the final manuscript.

## Pre-publication history

The pre-publication history for this paper can be accessed here:

http://www.biomedcentral.com/1472-6947/10/67/prepub
